# Pedicle Screw Pull-Out Strength Between the Jamshidi Needle and Pedicle Probe Techniques in an Osteoporotic Cancellous Bone Model: A Biomechanical Study

**DOI:** 10.7759/cureus.74747

**Published:** 2024-11-29

**Authors:** Shival Tharmaseelan, Mohd Hezery Harun, Fadzrul Abbas Mohamed Ramlee, Mohd Na'im Abdullah

**Affiliations:** 1 Orthopaedics and Traumatology, Universiti Putra Malaysia, Kuala Lumpur, MYS; 2 Aerospace Engineering, Universiti Putra Malaysia, Kuala Lumpur, MYS

**Keywords:** biomechanical model, jamshidi needle, pedicle probe, s: osteoporosis, spinal fusion surgery

## Abstract

Introduction

Spinal fusion surgery with pedicle screws is commonly performed to stabilize the spine of osteoporotic patients. However, securing a strong screw fixation in osteoporotic bone presents significant challenges due to the reduced bone density. This study aimed to compare the biomechanical performance in an osteoporotic bone model of pedicle screws inserted using two different techniques, the Jamshidi needle technique and the pedicle probe technique, as well as the influence of tapping on both these techniques. The research sought to determine if the surgical device used in aiding pedicle screw placement, pedicle probe (open technique) and Jamshidi needle (minimally invasive surgical (MIS) technique), affects the eventual stability of the screw in osteoporotic conditions. The findings of this study could enlighten surgical practices, potentially leading to improved clinical outcomes for patients suffering from osteoporosis-related spinal instability.

Materials and methods

An in vitro biomechanical comparative study was performed whereby pedicle screws were inserted into a standardized polyurethane foam model of grade 10, mimicking osteoporotic bone. Cylindrical poly-axial pedicle screws of 6.5 mm diameter and 45 mm length made out of medical-grade titanium alloy, Ti-6Al-4V, were inserted using four different techniques: Jamshidi needle, Jamshidi needle with tapping, pedicle probe, and pedicle probe with tapping. The screws were inserted in a standardized manner across all groups; the constructs were subsequently attached to the Material Testing System (MTS) 810 machine (MTS Systems Corporation, Eden Prairie, Minnesota, United States) using a customized jig. A direct-load-to-fail test was performed, where data was collected and tabulated into a force-displacement graph. The axial pull-out strength, axial stiffness, and displacement to failure of each construct were then extracted from the graph. Independent samples t-test was then used to compare and study the association between the groups.

Results

The pedicle probe technique demonstrated superior pull-out strength (698.36±16.34 N) compared to the Jamshidi needle technique (557.15±4.52N) (p<0.05). A greater displacement to failure was also seen in the pedicle probe group (2.26±0.04 mm) versus the Jamshidi needle group (1.18±0.06 mm) (p<0.05). However, the Jamshidi needle technique exhibited higher axial stiffness (336.88±23.24 N/mm) compared to the pedicle probe technique (208.82±7.82 N/mm) (p<0.05). In examining the influence of tapping on both techniques, results show significantly reduced pull-out strength and displacement to failure in the pedicle probe group.

Conclusions

The pedicle probe technique offers enhanced initial stability in the osteoporotic bone as evidenced by the superior pull-out strength and displacement to failure. On the other hand, the Jamshidi needle technique provides greater resistance to deformation, demonstrated by higher axial stiffness. Tapping should be carefully considered, especially while using the pedicle probe technique, as demonstrated by significantly reduced pull-out strength and displacement to failure. The choice of technique should be informed by specific clinical context balancing the need for initial stability, resistance to deformation, and risk of screw failure.

## Introduction

Lower back pain is a prevalent condition affecting millions of people worldwide, often leading to significant disability and reduced quality of life [1]. One common cause of lower back pain is spinal instability, which can result from various factors such as degenerative disc disease, trauma, or osteoporosis [2]. Spinal instability can lead to abnormal movement between vertebrae, causing pain and potential neurological deficits [3].

To address spinal instabilities, spinal fusion surgery is frequently performed, which involves the use of pedicle screws to stabilize the affected vertebrae [4]. Pedicle screws are threaded titanium or stainless-steel implants that are inserted into the vertebral pedicles to provide anchorage for rods and plates, thereby immobilizing the spine and promoting fusion [4]. These screws are essential for maintaining spinal alignment and stability during the healing process [4]. The treatment of spinal instability has advanced over time, and the recommended approach now is surgical spinal fusion. The fusion of the spinal segment using pedicle screws leads to limited or no flexibility at the enhanced motion segment. The introduction of pedicle screws can be attributed to Boucher in the 1950s, but their widespread use was established in the 1980s. Research demonstrated that pedicle screws were both practical and safer and had a higher success rate compared to the previously prevalent non-instrumented fusion.

Spinal fusion surgeries are commonly performed to treat conditions such as spinal deformities, spondylolisthesis, degenerative disc disease, and trauma [3]. The prevalence of conditions requiring spinal fusion surgery is significant, particularly among the elderly population, who are more prone to osteoporosis and degenerative spinal diseases [3]. Osteoporosis, a condition characterized by reduced bone density and quality, affects millions of people worldwide and increases the risk of fractures and spinal instability [5]. Consequently, the use of pedicle screws in osteoporotic patients is a common practice to ensure proper spinal stabilization and promote healing [5]. Therefore, pedicle screws play a pivotal role in achieving spinal fusion. However, their successful integration into osteoporotic bone presents challenges due to the diminished bone-screw interface and overall bone quality. Osteoporotic vertebrae exhibit decreased trabecular density, which compromises the stability of pedicle screws and leads to implant failure [6]. Studies have consistently shown that the rate of screw loosening is 1-15% in non-osteoporotic patients but up to 10-60% in osteoporotic subjects [6].

Despite their widespread use, pedicle screws are associated with several complications, including screw loosening, breakage, and malpositioning [5]. The accuracy and effectiveness of pedicle screw placement are critical for the success of these surgeries and for avoiding complications such as nerve root irritation, vascular injury, and the need for revision surgery [5]. Ensuring the optimal placement and fixation of pedicle screws is crucial to minimize these risks and improve surgical outcomes [5].

Therefore, various techniques have been developed to enhance the initial holding power and overall success of pedicle screw fixation. Traditionally, pedicle screws are inserted in an open procedure where a pedicle probe is used to aid screw placement [7]. The pedicle probe technique utilizes a probe to prepare the screw path, potentially offering better control and alignment during insertion. However, in recent times, minimally invasive surgical (MIS) techniques are gaining popularity as they are postulated to reduce tissue morbidity and improve patient recovery times [8]. Surgeons who prefer the MIS technique often use the Jamshidi needle to aid in the accurate placement of screws. The Jamshidi needle is used to create a pilot hole for the pedicle screw, which is believed to improve the screw's purchase in the bone [8].

Biomechanical studies play a vital role in understanding the performance and limitations of pedicle screws, particularly in osteoporotic bone models [8]. These studies evaluate various factors such as pull-out strength, axial stiffness, and displacement to failure, providing insights into the mechanical stability of different screw insertion techniques [8]. The pull-out strength of pedicle screws is a critical parameter, as it reflects the screw's ability to resist axial forces and maintain fixation within the bone [8].

In this study, we aim to assess and determine if the surgical device used in aiding pedicle screw placement, pedicle probe (open technique) and Jamshidi needle (MIS technique), affects the eventual stability of screw in osteoporotic conditions. By measuring the maximal pull-out strength, axial stiffness, and displacement to failure, this research seeks to provide valuable insights into the biomechanical performance of these insertions. Additionally, the study will investigate the impact of tapping on the maximal pull-out strength of pedicle screws in osteoporotic bone. The findings of this study could influence clinical practices and improve outcomes for patients undergoing spinal instrumentation in osteoporotic bone.

## Materials and methods

Pedicle screw instrumentation in synthetic cancellous bone model

Pull-out strength is a measure of the force required to dislodge a pedicle screw from the bone. It is a critical parameter for assessing the initial stability of the screw and its ability to withstand axial loads. Multiple studies have been performed with rigid polyurethane foam models to study the pull-out strength of pedicle screws [9]. The polyurethane foam model can mimic the microstructure of human trabecular bone and reduces the inter-specimen variability of bone mineral density commonly seen in cadaveric studies [9]. The synthetic polyurethane foam model (Model #1522-01, Pacific Research Laboratory Inc., Vashon Island, Washington, United States) was used in this study. The synthetic bone was supplied as a rectangular shape (test block) with grade 10 foam of density 160 kg/m^3^, which simulates a cadaveric spinal bone with osteoporosis. The foam type and their representative bone density are regulated and standardized as per the American Society for Testing and Materials (ASTM) F1839-08 protocol [10].

**Figure 1:**
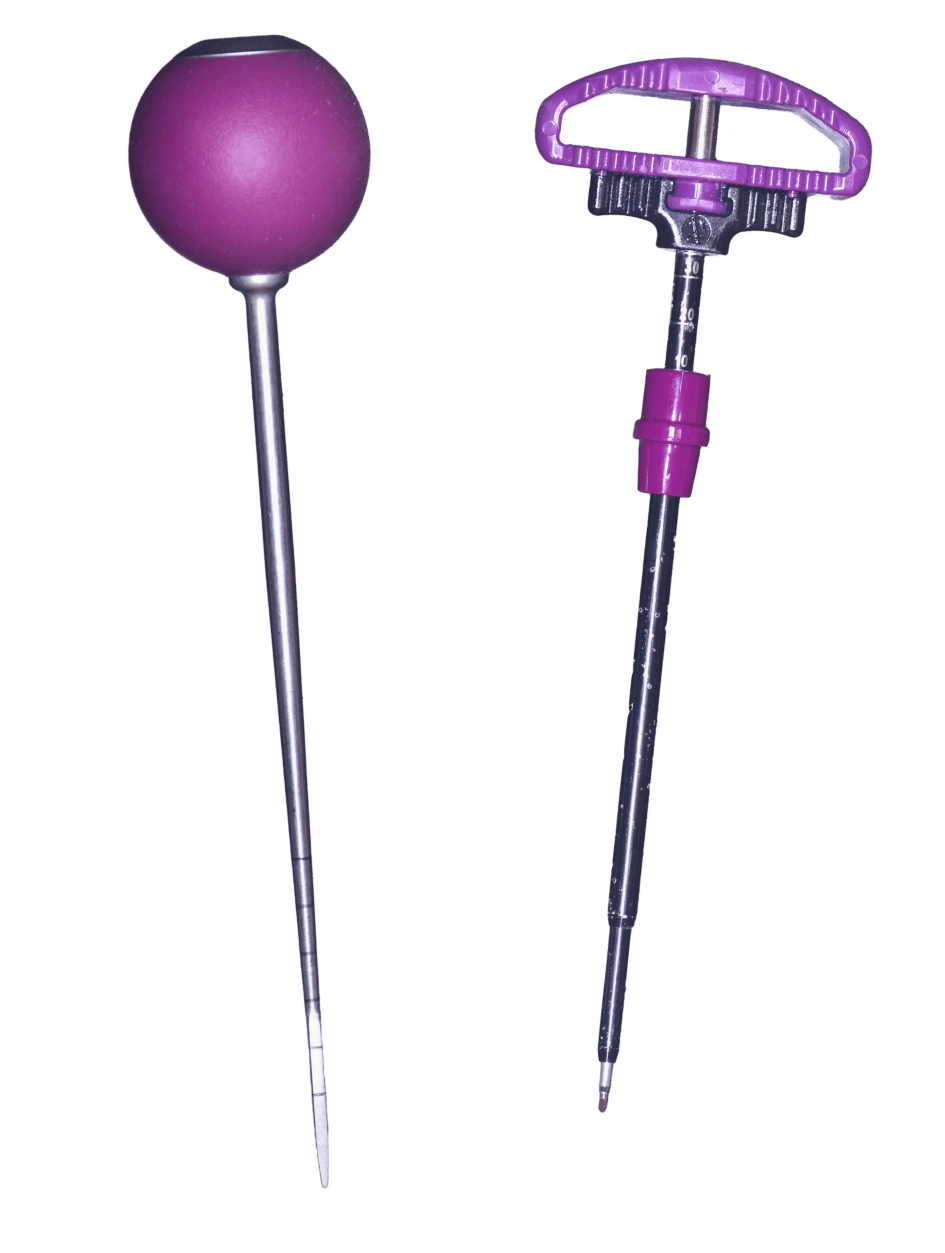
Pedicle probe (left) and Jamshidi needle (right)

Cylindrical poly-axial pedicle screws of 6.5 mm diameter and 45 mm length made out of medical-grade titanium alloy, Ti-6Al-4V, were used in the study. A pedicle probe or the Jamshidi needle (Figure 1) was used to create an initial tract prior to screw insertion. A 4.5-mm-diameter tap is used for the test groups that require tapping. This study involves four test groups (Table 1) where each test group is abbreviated by the device sequentially used for screw insertion: group 1: Jamshidi needle-guidewire insertion-screw insertion (JGS), group 2: Jamshidi needle-guidewire insertion-tapping-screw insertion (JGTS), group 3: pedicle probe-screw insertion (PS), and group 4: pedicle probe-tapping-screw insertion (PTS). In all groups, each screw was inserted to a consistent depth of 40 mm at a speed of 3 revolutions per minute by the same spine surgeon. A 40-mm-long screw was chosen as the anatomical range of pedicle chord length in the lumbar spine, according to literature, was found to be between 35 and 45 mm [11].

**Table 1:** Test groups and their respective abbreviations

Number	Name	Abbreviation
1	Jamshidi needle-guidewire insertion-screw insertion	JGS
2	Jamshidi needle-guidewire insertion-tapping-screw insertion	JGTS
3	Pedicle probe-screw insertion	PS
4	Pedicle probe-tapping-screw insertion	PTS

Pull-out strength testing

**Figure 2:**
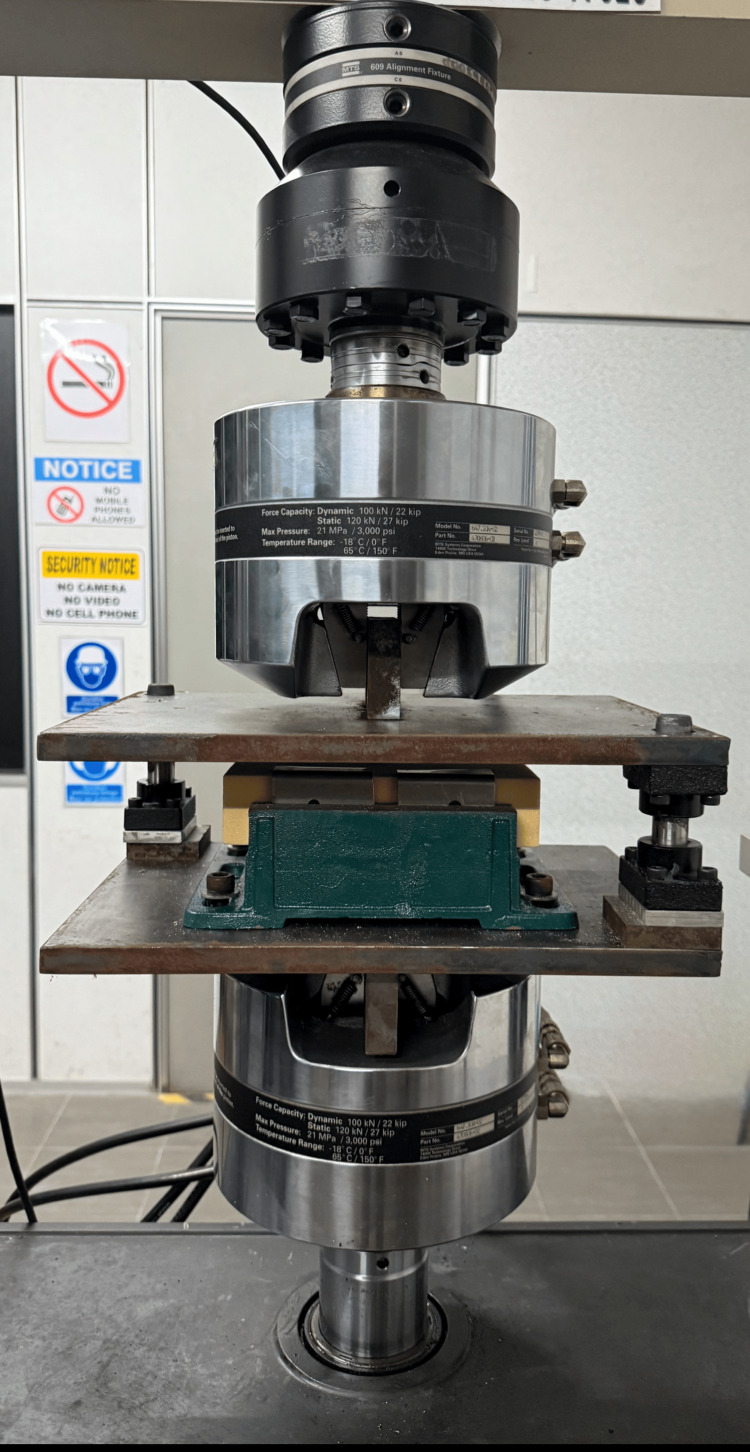
Apparatus and laboratory set-up for the pull-out test

The screw-foam construct was then mounted onto a customized jig (Figure 2) and tested as per the ASTM F543 protocol [12] on the Material Testing System (MTS) 810 machine (MTS Systems Corporation, Eden Prairie, Minnesota, United States). A tensile load of 15 kN was applied to the pedicle screw, and it was pulled at a rate of 5 mm per minute until the screw was released from the block. To ensure uniformity across specimens, the applied force of 15 kN was applied throughout the testing, and the pull rate of 5 mm per minute was maintained consistently for all samples. The pull-out strength testing was conducted three times for each test group.

The force-displacement data was constantly recorded at a sampling frequency of 50 Hz, and a force-displacement curve was obtained. The curve is then examined to calculate the maximum pull-out strength, axial stiffness, and displacement to failure. The maximal pull-out strength refers to the highest amount of tensile load (measured in Newtons) at which the screw becomes detached from the foam. Axial stiffness was determined by calculating the force needed to cause a unit of axial deformation upon the screw, given in N/mm², based on the slope of the linear segment of the curve. The displacement to failure, which is the distance in mm that the screw migrates before failure which is when the screw becomes detached from the foam, was obtained by measuring the x-axis of the linear section of the graph. The data was analyzed with IBM SPSS Statistics for Windows, Version 25.0 (Released 2017; IBM Corp., Armonk, New York, United States). All data were presented as mean±standard deviation for continuous and categorical data. Independent samples t-test was used to compare and study the association between groups where p<0.05 was considered statistically significant.

## Results

Biomechanical properties of the Jamshidi needle (JGS) and pedicle probe (PS)

Results of the present study indicate significant differences between the Jamshidi needle technique (JGS) and the pedicle probe technique (PS) across all measured variables in an osteoporotic bone model (Table 2). Specifically, the maximal pull-out strength was significantly higher with the PS technique, recording 698.36±16.34 N compared to 557.15±4.52 N with the JGS technique (p<0.05). In terms of axial stiffness, the JGS technique demonstrated superior performance, with a value of 336.88±23.24 N/mm, in contrast to 208.82±7.82 N/mm for the PS technique (p=0.0008). Additionally, the displacement to failure was greater with the PS technique, measuring 2.26±0.04 mm, while the JGS technique showed a displacement of 1.18±0.06 mm (p<0.05).

**Table 2:** Comparison of the biomechanical properties between JGS and PS JGS: Jamshidi needle-guidewire insertion-screw insertion; PS: pedicle probe-screw insertion

Variables	Technique		P-value	T-value
	JGS (mean±SD)	PS (mean±SD)		
Pull-out strength (N)	557.15±4.52	698.36±16.34	0.0001	-14.4295
Axial stiffness (N/mm)	336.88±23.24	208.82±7.82	0.0008	9.0458
Displacement to failure (mm)	1.182±0.06	2.26±0.04	0.00001	-27.0053

Impact of tapping in the Jamshidi needle group

In examining the impact of tapping on the maximal pull-out strength of the pedicle screw inserted via the Jamshidi needle technique (JGS) compared to the Jamshidi needle technique with tapping (JGTS) in an osteoporotic bone model, the results revealed nuanced differences (Table 3). The pull-out strength for the JGS group was 557.15±4.52 N, while the JGTS group demonstrated a slightly lower value of 538.89±10.75 N, with a p-value of 0.053, indicating a marginal, non-significant reduction due to tapping. For axial stiffness, a significant decrease was observed with tapping. The JGS group exhibited a stiffness of 336.89±23.24 N/mm, compared to 256.62±4.61 N/mm for the JGTS group, with a p-value of 0.004. However, for displacement to failure, the difference was not statistically significant, with the JGS group recording 1.18±0.06 mm and the JGTS group 1.25±0.06 mm and a p-value of 0.244.

**Table 3:** Impact of tapping in the Jamshidi needle group JGS: Jamshidi needle-guidewire insertion-screw insertion; JGTS: Jamshidi needle-guidewire insertion-tapping-screw insertion

Variables	Technique		P-value	T-value
	JGS (mean±SD)	JGTS (mean±SD)		
Pull-out strength (N)	557.15±4.52	538.89±10.75	0.053	2.7125
Axial stiffness (N/mm)	336.89±23.24	256.62±4.61	0.004	5.8683
Displacement to failure (mm)	1.18±0.06	1.25±0.06	0.244	-1.3661

Impact of tapping in the pedicle probe group

In assessing the impact of tapping on the maximal pull-out strength of the pedicle screw inserted via the pedicle probe technique (PS) compared to the pedicle probe technique with tapping (PTS) in an osteoporotic bone model, notable differences were observed. The pull-out strength for the PS group was 651.35±8.62 N, while the PTS group showed a significantly lower strength of 538.89±10.75 N, with a p-value of 0.012, indicating a substantial reduction in pull-out strength due to tapping. However, the axial stiffness remained unchanged between the two groups, with both recording 208.82±7.82 N/mm and a p-value of 0.200, suggesting no significant effect of tapping on stiffness. For displacement to failure, the PS group exhibited a value of 2.26±0.04 mm, compared to 2.10±0.04 mm in the PTS group, with a p-value of 0.008, indicating a statistically significant decrease in displacement to failure with tapping. These findings suggest that while tapping significantly reduces pull-out strength and displacement to failure in the pedicle probe technique, it does not significantly affect axial stiffness.

**Table 4:** Impact of tapping in the pedicle probe group PS: pedicle probe-screw insertion; PTS: pedicle probe-tapping-screw insertion

Variables	Technique		P-value	T-value
	PS (mean±SD)	PTS (mean±SD)		
Pull-out strength (N)	698.36±16.34	651.35±8.62	0.012	-4.41
Axial stiffness (N/mm)	208.82±7.82	208.82±7.82	0.200	1.53
Displacement to failure (mm)	2.26±0.04	2.10±0.04	0.008	-4.96

## Discussion

Biomechanical properties of the Jamshidi needle (JGS) and pedicle probe (PS)

The current study highlights important differences between the Jamshidi needle technique (JGS) and the pedicle probe technique (PS) in an osteoporotic bone model. The results of this study indicate significant differences between the Jamshidi needle technique and the pedicle probe technique across all measured variables in an osteoporotic bone model. Specifically, the maximal pull-out strength was significantly higher with the PS technique, recording 698.36±16.34 N compared to 557.15±4.52 N with the JGS technique (p<0.05). These results differ from similar studies performed on human cadaveric specimens where there was no significant difference between the two techniques [7]. Our study shows the potential benefit of using a larger entry tract for screw insertion in osteoporotic bones in terms of pull-out strength. The larger entry tract created by the PS technique likely facilitates a more substantial engagement with the surrounding bone, thereby enhancing screw stability and pull-out resistance [13]. Moreover, the findings align with Siasios et al. [14], who reported that the mechanical properties of the screw-bone interface are significantly influenced by the size and shape of the pilot hole. A larger entry tract, such as that created by the PS technique, enhances the screw's ability to resist axial forces, thereby improving the overall durability of the fixation.

In terms of axial stiffness, the JGS technique demonstrated superior performance, with a value of 336.88±23.24 N/mm, in contrast to 208.82±7.82 N/mm for the PS technique (p=0.001). This result suggests that the JGS technique may provide better initial stability, which is crucial for maintaining spinal alignment and stability during the healing process. Previous studies have shown that higher axial stiffness is associated with improved mechanical stability and reduced risk of screw loosening as it is able to provide better resistance to deformation under axial loads [15].

Additionally, the displacement to failure was greater with the PS technique, measuring 2.26±0.04 mm, while the JGS technique showed a displacement of 1.18±0.06 mm (p<0.05). This finding indicates that the PS technique allows for greater displacement before failure, which may be beneficial in accommodating the compromised bone structure in osteoporotic patients.

In summary, the PS technique provides superior pull-out strength and displacement to failure, while the JGS technique offers better axial stiffness. These findings suggest that the choice of insertion technique should be tailored to the specific needs of the patient and the clinical scenario. For instance, in cases where initial stability is paramount, the JGS technique may be preferred due to its higher axial stiffness. Conversely, in situations where greater displacement to failure is desired, the PS technique may be more suitable.

Impact of tapping in the Jamshidi needle group

When examining the impact of tapping with the Jamshidi needle technique, tapping led to a slight reduction in pull-out strength and a significant decrease in axial stiffness, though it did not significantly affect displacement to failure.

The impact of tapping in the Jamshidi needle group appears to be limited for axial stiffness where a significant decrease was seen with the JGS group exhibiting a stiffness of 336.89±23.24 N/mm, compared to 256.62±4.61 N/mm for the JGTS group, with a p-value of 0.004. This result aligns with findings from the literature which reported that tapping can reduce the axial stiffness of pedicle screws by weakening the bone around the screw, thereby decreasing the overall stability of the screw-bone construct [16]. The reduction in axial stiffness due to tapping may be attributed to the removal of bone material during the tapping process, which can lead to a less dense and less supportive bone environment for the screw. Secondly, for the pull-out strength in the Jamshidi needle group, there was a marginal, non-significant reduction due to tapping with a p-value of 0.053. This finding is consistent with previous studies that have shown tapping can reduce the pull-out strength of pedicle screws, particularly in osteoporotic bone. The reduction in pull-out strength due to tapping can be attributed to the creation of microfractures and reduced bone density around the screw, which weakens the bone-screw interface [16]. However, for displacement to failure, the difference was not statistically significant with a p-value of 0.244. This suggests that while tapping may affect the initial stability parameters such as pull-out strength and axial stiffness, it does not significantly impact the displacement to failure. This finding is supported by studies that have shown displacement to failure is more influenced by the overall bone quality and screw design rather than the tapping process itself [17]. While initial stability parameters such as pull-out strength and axial stiffness appear to be significantly influenced by tapping, it does not impact the displacement to failure.

The results of this study can be interpreted as evidence that tapping while using the Jamshidi needle may not be beneficial for enhancing the pull-out strength and axial stiffness of pedicle screws in osteoporotic bone. Instead, tapping appears to marginally reduce these parameters, and hence in clinical scenarios involving osteoporotic bone, avoiding tapping may be advantageous for maintaining higher pull-out strength and axial stiffness.

Impact of tapping in the pedicle probe group

With the pedicle probe technique, tapping resulted in a marked reduction in pull-out strength and in displacement to failure, with no substantial impact on axial stiffness. Tapping significantly reduced pull-out strength in the pedicle probe technique group (PTS) compared to the non-tapped group (PS) with a p-value of 0.012. Inceoglu et al. state that the additional stress created from tapping may further weaken the osteoporotic bone leading to lower pull-out strength [15]. The pedicle probe technique did not show a significant change in axial stiffness (p=0.200) with tapping, suggesting that this technique may be more resilient to the effects of tapping on stiffness. Lastly, displacement to failure results showed that tapping significantly reduced displacement (p=0.008) indicating a less flexible and more brittle bone-screw interface. This reduction in displacement to failure can be detrimental in clinical scenarios, as it may lead to a higher risk of screw loosening and failure under physiological loads [16].

Clinical significance of the study

The present study revealed significant differences in pull-out strength, axial stiffness, and displacement to failure when comparing the Jamshidi needle technique and the pedicle probe technique, with and without tapping. The superior pull-out strength and displacement to failure of the non-tapped pedicle probe technique suggest that it may be a more reliable method for pedicle screw insertion in osteoporotic bone. This technique can potentially reduce the risk of screw failure postoperatively, leading to better surgical outcomes and patient satisfaction.

The impact of tapping on the biomechanical performance of pedicle screws highlights the need for careful consideration of this step during surgery. The above results indicate that while tapping generally reduces mechanical performance in both techniques, the extent and nature of its effects vary between the two methods. While tapping may facilitate screw insertion, it can also compromise the bone-screw interface, particularly in osteoporotic bone. Surgeons should weigh the benefits and drawbacks of tapping based on the specific clinical scenario and patient bone quality. Avoiding tapping or using under-tapping techniques may enhance the initial holding power and stability of pedicle screws in osteoporotic patients [16,18]. The findings suggest that avoiding tapping may enhance the pull-out strength and stability of pedicle screws, particularly in osteoporotic bone.

Future studies should further investigate the long-term clinical outcomes of these techniques to validate their efficacy and safety in osteoporotic patients. Similar studies can also be replicated using cancellous bone models with normal bone density to see if the result patterns obtained in this study are also seen in healthy bone or otherwise.

Limitations of the study

This study has several limitations that should be considered when interpreting its results. Firstly, the use of a synthetic osteoporotic bone model offers a controlled testing environment but does not fully replicate the complexity of human osteoporotic bone. Human bones exhibit a wide range of microarchitectural properties and densities, which can affect implant performance. Therefore, the findings may not fully reflect screw behavior in actual clinical scenarios, where bone quality can vary significantly.

Secondly, the study focused on initial mechanical parameters, such as pull-out strength, axial stiffness, and displacement to failure, which are important for understanding immediate screw stability but do not capture long-term outcomes like screw loosening, migration, or patient-reported outcomes. These factors are critical for evaluating the real-world durability and effectiveness of pedicle screws in osteoporotic patients, and future studies should include long-term follow-ups to assess these outcomes.

Finally, the study did not account for important variables that have previously been reported in the literature as potential factors that affect the pull-out strength of pedicle screws such as screw design, insertion angle, or bone cement augmentation, all of which can significantly affect screw stability. Investigating these factors would offer a more comprehensive understanding of screw performance.

While this study provides valuable insights into the initial biomechanical performance of pedicle screws, future research should address these limitations to validate the findings and better understand the long-term success of pedicle screw implantation in osteoporotic patients.

## Conclusions

The present study demonstrates that the pedicle probe technique (PS) offers superior pull-out strength and greater displacement to failure compared to the Jamshidi needle technique (JGS) in an osteoporotic bone model, suggesting that the PS technique may provide enhanced initial stability and flexibility under load. However, the JGS technique outperforms the PS technique in terms of axial stiffness, indicating that it offers greater resistance to deformation under applied forces. Tapping, when applied to the JGS technique, resulted in a marginal reduction in pull-out strength and a significant decrease in axial stiffness while having no significant effect on displacement to failure. Conversely, tapping in the PS technique significantly reduced both pull-out strength and displacement to failure, though axial stiffness remained unchanged. These findings underscore the need to carefully consider the biomechanical properties of each screw insertion technique in osteoporotic bone, particularly when deciding whether to use tapping. The choice of technique and the use of tapping should be informed by the specific clinical context, balancing the need for initial stability, resistance to deformation, and the risk of screw loosening or failure.
